# Using Discriminant Analysis to Predict Frailty in Community-Dwelling Older Adults in Taiwan

**DOI:** 10.1097/jnr.0000000000000716

**Published:** 2025-12-26

**Authors:** Meei-Horng YANG, Hung-Ru LIN, Chieh-Yu LIU, Liang-Kung CHEN, Tzu-Ying LEE, Kee-Hsin CHEN

**Affiliations:** 1 Department of Nursing, Landseed International Hospital, Taoyuan, Taiwan, ROC; 2 Department of Nursing, National Defense Medical Center, Taipei, Taiwan, ROC; 3 Department of Nursing, Yuanpei University of Medical Technology, Hsinchu, Taiwan, ROC; 4 School of Nursing, National Taipei University of Nursing and Health Sciences, Taipei, Taiwan, ROC; 5 Biostatistical Consultant Lab., Institute of Community Health Care, College of Nursing, National Yang Ming Chiao Tung University, Taipei, Taiwan, ROC; 6 Center for Geriatrics and Gerontology, Taipei Veterans General Hospital, Taipei, Taiwan, ROC; 7 Center for Healthy Longevity and Aging Sciences, National Yang Ming Chiao Tung University, Taipei, Taiwan, ROC; 8 Taipei Municipal Gan-Dau Hospital (Managed by Taipei Veterans General Hospital), Taipei, Taiwan, ROC; 9 Post‑Baccalaureate Program in Nursing, College of Nursing, and Cochrane Taiwan, Taipei Medical University, Taipei, Taiwan, ROC; 10 Department of Nursing, and Research Center in Nursing Clinical Practice, Wan Fang Hospital, Taipei Medical University, Taipei, Taiwan, ROC; 11 Faculty of Health and Medical Sciences, School of Medicine, Taylor’s University, Subang Jaya, Malaysia

**Keywords:** frailty, phenotype, older adults, prediction, discriminant analysis

## Abstract

**Background:**

The definition of frailty is still debated, resulting in the development of various measurement tools. Having a convenient and accurate frailty screening instrument is essential to providing appropriate care to community-dwelling older adults in terms of facilitating the delayed onset of frailty and preventing disability.

**Purpose:**

This study was conducted to develop a simple, convenient, and rapid screening method for frailty classification in community-dwelling older adults that incorporates the most significant predictive factors from the Study of Osteoporotic Fractures index components and the Kihon Checklist tool domains.

**Methods:**

Convenience sampling was used to gather longitudinal data from 110 community-dwelling older adults at baseline (T0), 6 months (T1), and 1 year (T2) using three different frailty screening tools. The Fried frailty phenotype tool was used as the gold standard. Linear discriminant analysis was conducted to create an effective model for accurately classifying frailty states.

**Results:**

The discriminant analysis generated three statistical significant functions, which respectively explained 33.6% (*Rc*=.58; *df*=3; *p*<.0001), 26.0% (*Rc*=.51; *df*=2; *p*<.0001), and 29.2% (*Rc*=.54; *df*=2; *p*<.0001) of the predictive power of prefrail/frail risk. The discriminant functions demonstrated sensitivities of 64.6%–69.4% for identifying the prefrail/frail group and specificities of 77.1%–90.9% for identifying the robust group. The developed method successfully classified the correct robust and prefrail/frail states for 71.6%–79.1% of participants. The findings of this longitudinal study show weight loss, reduced energy levels, physical function, activities of daily living (IADL lifestyle), and eating function to be the most significant factors at baseline for accurately classifying community-dwelling older adults into robust and prefrail/frail states over a 1-year follow-up period.

**Conclusions/Implications for Practice:**

Eating function was identified as the strongest factor of influence on the correct prediction of frailty status. Nurses may use the five questionnaire-based domains in initial assessments to classify frailty in community-dwelling older adults with a 1-year accuracy of at least 70%. Those identified as at-risk should be referred to physicians, nutritionists, rehabilitation specialists, and/or long-term care services to optimally delay or prevent the onset of frailty in this population.

## Introduction

Although the definition of frailty remains controversial, it is generally recognized as a gradual age-related progressive decline in physiological systems that leads to impaired reserves of intrinsic capacity. The result of frailty is extreme vulnerability to stressors and increased risk of adverse health outcomes (World Health Organization, 2015) such as falls (Doñate-Martínez et al., 2022; Yang et al., 2023), disability (Doñate-Martínez et al., 2022), hospitalization (Chang et al., 2018), and death (Hwang et al., 2023). Frailty, although often overlapping with comorbidity and disability, is its own unique condition. In terms of their relation to frailty, comorbidity is a risk factor and disability is a consequence (Fried et al., 2001). All older adults are at risk of developing frailty (Hoogendijk et al., 2019).

A meta-analysis of studies covering 62 countries revealed the prevalence of prefrailty and frailty among community-dwelling individuals over 50 years old to be 46% and 12%, respectively (O’Caoimh et al., 2021). The prevalence of frailty among community-dwelling older adults in Asia has been reported as 20.5% (To et al., 2022). Data from Taiwan’s Survey of Health and Living Status of the Elderly indicate that the prevalence of prefrailty and frailty among community-dwelling older adults in Taiwan is, respectively, 40% and 4.9% (C.-Y. Chen et al., 2010). A meta-analysis by Qin et al. (2023) identified demographic factors (e.g., age, female gender, education, income, and living alone), health-related factors (e.g., self-perceived health, cognitive function, comorbidity, and polypharmacy), and physical factors (e.g., activities of daily living [ADL] and instrumental activities of daily living [IADL] limitations) as risk factors for the development of frailty in community-dwelling older adults. The findings of Yuan et al. (2022) suggest that better understanding the factors related to frailty can aid in its prevention. When analyzing frailty predictors, considering temporal correlations can provide additional insights. Tchalla et al. (2023) highlighted that in a 75-month study with seven follow-up assessments of community-dwelling older adults, cognitive impairment and dementia emerged as significant predictors of worsening frailty trajectories after adjusting for age and gender. Moreover, hypertension was identified as a key discriminating factor influencing frailty trajectories, specifically in the transition from frail to prefrail states and from prefrail to frail states.

In the literature review conducted by Buta et al. (2016), 67 different tools were identified for assessing frailty. One of these in particular, the Fried frailty phenotype (FP), covering the five components of weight loss, exhaustion, slowness, weakness, and low activity (Fried et al., 2001), is widely regarded as the gold standard for frailty assessment (Ambagtsheer et al., 2020). The Study of Osteoporotic Fractures (SOF) index, a simplified version of the FP tool, was initially designed to predict the risks of falls, disability, fractures, and death, and offers a useful definition of frailty to identify older women at risk of adverse health outcomes in clinical practice. This index comprises three components: weight loss, lower extremity function, and reduced energy level. Due to its ease of use, the SOF tool is applicable in community settings as well as clinical practice (Ensrud et al., 2008; Kiely et al., 2009). The 25-item Kihon Checklist (KCL) addresses seven domains, including (1) activities of daily living, (2) physical function, (3) nutrition, (4) eating function, (5) socialization, (6) memory, and (7) mood. In addition to assessing frailty levels (Jang et al., 2017), the KCL may be used in evaluating the effectiveness of frailty intervention programs (Sewo Sampaio et al., 2016).

Taiwan, which became an “aging society” in 1993 and “aged society” in 2018, is currently experiencing rapid population aging. According to the population projections of the National Development Council, Taiwan, ROC (2024), Taiwan will meet the definition of a “superaged society” by 2025, when older adults should account for 20% of the total population, and an “ultra-aged society” by 2031, when this ratio is expected to reach 25%. Due to the long-term impact of low fertility rates, the pace of population aging in Taiwan is projected to be faster than in other major countries, surpassing Japan by 2047 and remaining slightly lower than South Korea by 2070.

In 2017, the Taiwan government launched the second 10-year Long-Term Care Plan (long-term care 2.0), under which substantial funds have been invested in promoting and implementing preventive measures. Considering setting and manpower limitations, SOF and KCL were the tools approved under this plan as frailty screening tools. To the best of our knowledge, no longitudinal studies have been conducted to investigate, using FP as the gold standard for frailty, which SOF components and KCL domains most accurately identify robust and prefrail/frail states in community-dwelling older adults.

Therefore, this study was designed to investigate how compositions from KCL domains and SOF components at baseline, 6 months, and 1 year in community-dwelling older adults, and compare results against those generated by the FP tool. The 6-month follow-up duration aligns with the evidence-based literature showing that interventions at least 5 months in duration are effective in improving frailty outcomes in older adults (Theou et al., 2011). Given that SOF and KCL are commonly used in Taiwan for frailty screening but are overly time-consuming to be generally used in community settings, this study seeks to identify a streamlined subset of screening items that maintains adequate precision. The findings of the study are expected to serve as a reference for decision-making in frailty risk assessment and care strategy development for the community-dwelling older adult population.

## Methods

### Participants and Study Design

A longitudinal study design was used, and patients aged 65 years or older in Miaoli County, Taiwan, were referred to the researchers by one outpatient clinic, six public health centers, and six branches of a social welfare foundation between March 2021 and March 2022. Inclusion criteria were (1) community-dwelling individuals aged 65 or older; (2) able to communicate in Mandarin, Taiwanese, or Hakka; (3) willing to undergo frailty assessments using the screening tools at three different times; and (4) signing written informed consent to participate. Exclusion criteria were (1) currently hospitalized or residing in a nursing home; (2) diagnosed with dementia; (3) bedridden or in the terminal stage of illness; (4) taking medication for Alzheimer’s disease or antidepressants; and (5) having a history of stroke or upper/lower limb surgery within the past 3 months.

The minimum sample size was calculated using G-power 3.1, employing repeated-measures statistical methods. The medium effect size assumption of Cohen (1988) was adopted. The effect size was set at 0.15, with an alpha level (α) of .05 and a power of 0.8. The study involved three measurement time points. The calculated minimum sample size was 88 participants. Considering an anticipated attrition rate of 20%, the target sample size was adjusted to 110 participants. The study objectives and procedures were thoroughly explained to the participants by a single researcher. After the participants signed informed consent, the researcher conducted one-on-one interviews, reading each question aloud and performing frailty measurements to complete data collection.

### Measures

Frailty was assessed using the Fried frailty phenotype (FP), Study of Osteoporotic Fractures (SOF), and Kihon Checklist (KCL) instruments at baseline (T0), 6 months (T1), and 12 months (T2). With the exception of measuring slowness, weakness, and physical activity directly for the FP instrument, all other components of the FP tool, as well as the SOF and KCL tools, gathered information via questionnaires conducted during individual, face-to-face interviews. The measurement outcomes were categorized as robust, prefrail, or frail. In the analysis, participants categorized as either prefrail or frail were included in the same group.

#### Fried Frailty Phenotype (FP)

The FP tool (Fried et al., 2001) covers the five phenotypic components of weight loss, exhaustion, slowness, weakness, and low activity. Unintentional weight loss is defined as a loss of either 5% body weight or >3 kg during the past 1-year period. Exhaustion is defined as feeling tired or lacking energy for >3 days during the past week. Slowness is measured by walking a distance of 5 m at a normal pace, with the best time out of two trials recorded and adjustments made for height and gender, with the lowest 20% defined as having slowness. Weakness is measured using a TTM-YD electronic grip strength device, with the best value of two dominant-hand trials recorded in kilograms and adjustments made for gender and body mass index (calculated as weight [kg]/height [m^2^]), with the lowest 20% defined as having weakness. For low activity, the Taiwan International Physical Activity Questionnaire Short Form was used with permission from the Health Promotion Administration of Taiwan’s Ministry of Health and Welfare to calculate the number of days and the duration of physical activity with different intensities during the past week and to convert this information into calorie expenditure (L.-J. Chen et al., 2014). Participants whose calorie expenditure fell within the lowest 20% for their gender were defined as having low activity. Finally, frailty state was categorized as robust (0 components), prefrail (1–2 components), or frail (3–5 components).

The Taiwanese version of the International Physical Activity Questionnaire Short Form was previously shown to have a content validity index of .99, with the language equivalence and meaning similarity between the English and Chinese versions also .99 and an intraclass correlation coefficient of .70 (L.-J. Chen et al., 2014). The FP provides a minimum 3-year predictive validity for adverse outcomes such as falls, hospitalization, disability, and mortality in older adults, with hazard ratios ranging from 1.82 to 4.46 (Fried et al., 2001). In this study, the Cronbach’s α for the FP tool was .57, which is below the threshold of .7 proposed by Nunnally (1978). As scales with fewer than 10 items tend to have lower Cronbach’s α values, inter-item correlations were calculated, resulting in a value of .2, which falls within the optimal range of .2 to .4 recommended by Briggs and Cheek (1986).

#### Study of Osteoporotic Fractures (SOF)

The SOF tool developed by Ensrud et al. (2008) includes three components: (1) Weight loss: Unintentional weight loss of 5% or more or of 3 kg or more during the previous year. (2) Lower extremity function: Ability to rise from a chair five times without using one’s arms for support. (3) Reduced energy level: Feeling for >3 days during the past week that everything is an effort. Frailty state is categorized in the SOF into robust (0 components), prefrail (1 component), and frail (2 or more components). The SOF tool has demonstrated concurrent validity with the FP, as evidenced by a Spearman rank correlation coefficient of .51 and a weighted kappa of .47, indicating a significant moderate correlation (Hu et al., 2019). In this study, as the SOF tool contains only three items, the Cronbach’s α value was .35 and the inter-item correlations were .14, both of which fall below the acceptable level for internal reliability.

#### Kihon Checklist (KCL)

The KCL was designed as a frailty and disability screening tool by a Japanese Ministry of Health, Labor and Welfare research group in 2006. It consists of 25 yes/no items in seven domains: (1) activities of daily living (IADL lifestyle), (2) physical function, (3) nutrition, (4) eating function, (5) socialization, (6) memory, and (7) mood. The total score ranges from 0 to 25, with a higher score indicating a greater degree of frailty (Sewo Sampaio et al., 2016). Frailty state score ranges are 0–3 for robust, 4–7 for prefrail, and ≥8 for frail (Satake et al., 2016). The Taiwanese version of the KCL (Hsieh, 2016) was used in this study, which has an internal consistency Cronbach’s α of .7, test-retest reliability with intraclass correlation coefficient values >.6, and content validity index values ranging from .76 to 1.0.

### Ethical Considerations

This study was reviewed and approved by the institutional review board (IRB: 120711) of a medical center in central Taiwan. All of the participants provided written informed consent before enrollment in the study.

### Data Analysis

First, at baseline, the three components of the SOF frailty screening tool (1) weight loss, (2) lower extremity function and (3) reduced energy level, along with the seven domains of the KCL frailty screening tool (1) IADL lifestyle, (2) physical function, (3) nutrition, (4) eating function, (5) socialization, (6) memory, and (7) mood, were used as 10 predictors to examine the screening accuracy of the gold-standard FP at T0, T1, and T2. Discriminatory analysis involves creating a classification function based on subjects with clearly defined categories with the goal of minimizing misclassification errors, allowing the function to accurately determine the category of new subjects. Before performing the discriminant analysis in this study, the correlation matrix was examined to check for multicollinearity, ensuring that the variables were not highly intercorrelated. Discriminant analysis using a two-group stepwise procedure was conducted to determine the degree to which the 10 selected predictors accurately classify robust and prefrail/frail.

Second, linear discriminant analysis was employed to identify different generalized patterns of frailty to indicate differences between the two groups. Specifically, three discriminant functions were identified as significant for optimal discrimination between the two groups. This approach takes advantage of the common variance among the individual aspects or frailty state items. Pearson’s correlation coefficients were computed to evaluate potential associations among the predictors used, with a value of *p*<.05 considered to indicate statistical significance. All analyses were conducted using IBM SPSS Statistics 20.0 (IBM Corp., Armonk, NY, USA).

## Results

### Participant Characteristics and Frailty States

One hundred and ten older adults living in the community completed the frailty screening, with results analyzed across three time points: T0, T1, and T2. Participant attrition over the study period was due to several factors, including admission to an assisted living facility or dementia group home, death, imprisonment, refusal to participate, and loss of follow-up after multiple contacts. Fifteen participants were lost at T1 and 13 at T2. Consequently, the study tracked 110 participants at T0, 95 at T1, and 97 at T2.

The participants were recruited from three primary venue types: six Tier C-LTC (Long-term Care Plan 2.0) stations (32.7%), six angel stations of a nonprofit welfare foundation (32.7%), and one outpatient clinic (34.6%). The baseline characteristics and frailty states of participants as measured using the FP tool are presented in Table 1, with 55 classified as robust and 55 as prefrail/frail at T0. The mean ages (*SD*s) were 74.8 (6.1) years for the robust group and 77.6 (7.7) years for the prefrail/frail group. Notably, 56.4% of the robust group were aged 65–74 years, while 47.3% of the prefrail/frail group were aged 75–84 years. Gender distribution revealed a higher proportion of women in both groups at 54.5% in the robust group and 58.2% in the prefrail/frail group.

**Table 1:** Baseline Characteristics and Frailty State Measured Using the FP Tool Among Participants (N=110)

Characteristic	*n* (%)	
	Robust (*n*=55)	Prefrail/Frail (*n*=55)
Age (year, mean and *SD*)	74.8 (6.1)	77.6 (7.7)
Age		
65–74	31 (56.4)	19 (34.5)
75–84	22 (40.0)	26 (47.3)
≥85	2 (3.6)	10 (18.2)
Gender		
Female	30 (54.5)	32 (58.2)
Male	25 (45.5)	23 (41.8)
Educational level (years)		
≤6	26 (47.3)	36 (65.5)
>6	29 (52.7)	19 (34.5)
Marital status		
Married with partner	32 (58.2)	18 (32.7)
Widowed/divorced/single	23 (41.8)	37 (67.3)
Living status		
Living accompanied	40 (72.7)	32 (58.2)
Living alone	15 (27.3)	23 (41.8)
Satisfaction with the environment		
Very satisfied/satisfied	44 (80.0)	35 (63.6)
Neutral	11 (20.0)	15 (27.3)
Dissatisfied/very dissatisfied	0 (0)	5 (9.1)
Self-rated health		
Good	21 (38.2)	4 (7.3)
Fair	31 (56.4)	32 (58.2)
Poor	3 (5.5)	19 (34.5)
Living community		
Urban	23 (41.8)	27 (49.1)
Township	32 (58.2)	28 (50.9)
Ethnicity		
Non-Hakka	25 (45.5)	23 (41.8)
Hakka	30 (54.5)	32 (58.2)
Main source of finances		
Self and spouse’s pension/savings/rental investment income/working income	26 (47.3)	22 (40.0)
Social assistance/national and older annuities/agriculture and fishery insurance	13 (23.6)	21 (38.2)
Dependent (child or grandchild)	16 (29.1)	12 (21.8)
Living expense (NTD/month)		
<6K	22 (40.0)	20 (36.4)
6–11K	17 (30.9)	25 (45.5)
≥12K	16 (29.1)	10 (18.2)

*Note.* NTD=New Taiwan dollar.

Educational level showed the majority in the robust group had more than 6 years of education (52.7%), while 65.5% in the prefrail/frail group had 6 years or less. In terms of marital status, most of the robust participants were married (58.2%), while the majority in the prefrail/frail group were widowed, divorced, or living alone (67.3%). The majority of participants did not live alone, with 72.7% in the robust group and 58.2% in the prefrail/frail group living with others. Home environment satisfaction was high in both groups: 80.0% in the robust group and 63.6% in the prefrail/frail group. Self-perceived health was predominantly fair in both groups, with 56.4% in the robust group and 58.2% in the prefrail/frail group.

Regarding area of residence, a majority resided in townships: 58.2% in the robust group and 50.9% in the prefrail/frail group. Ethnicity distribution was similar between the groups, with ethnic Hakkas comprising 54.5% of the robust group and 58.2% of the prefrail/frail group. The main financial source for both groups was pension, savings, rental income, or work income: 47.3% in the robust group and 40.0% in the prefrail/frail group. Living expenses varied, with 40.0% of the robust group having expenses below 6,000 NTD/month and 45.5% of the prefrail/frail group having expenses between 6,000 and 11,000 NTD/month.

### Discriminant Analysis

The Pearson correlation coefficients for the study predictors are shown in Table 2. Before conducting the analysis, the pooled within-groups correlation matrix was examined for high correlations among the predictor variables. Correlations among the stability scores ranged from −.06 to .58 and with weight loss from −.06 to .41; stability scores and reduced energy level were correlated from .05 to .58. In terms of correlations with stability scores, IADL ranged from −.14 to .51, physical function ranged from −.06 to .51, and eating function ranged from −.06 to .40. These weak correlations support the independence of the variables and the validity of including each variable in the discriminant analysis.

**Table 2:** Pearson’s Correlation Coefficients for the Predictors Used in the Discriminant Analysis at T0 (N=110)

Predictor	1	2	3	4	5	6	7	8	9	10
1. Weight loss	1.00									
2. Lower extremity function	−.19	1.00								
3. Reduced energy level	.05	.17	1.00							
4. IADL lifestyle	−.14	.39	.34	1.00						
5. Physical function	−.06	.47	.35	.51	1.00					
6. Nutrition	.41	.07	.07	.00	.17	1.00				
7. Eating function	−.06	.21	.42	.25	.37	.03	1.00			
8. Socialization	.06	.06	.36	.31	.32	.10	.24	1.00		
9. Memory	.03	.16	.27	.15	.09	.07	.14	.14	1.00	
10. Mood	.06	.08	.58	.35	.32	.13	.40	.42	.36	1.00

*Note.* IADL lifestyle = activities of daily living domain in the Kihon Checklist.

The stepwise discriminant analysis (Table 3) revealed high statistical significance using Wilks’ λ to test the discriminant function. At T0, Wilks’ λ=.66, χ^2^ (*df*=3)=43.68, and *p*<.0001. At T1, Wilks’ λ=.74, χ^2^ (*df*=2)=27.61, and *p*<.0001. At T2, Wilks’ λ=.71, χ^2^ (*df*=2)=31.88, and *p*<.0001. The discriminant function at T0 included three predictors: weight loss, reduced energy level, and physical strength. At T1, the predictors were IADL lifestyle and eating, and at T2, the predictors were physical strength and eating. The eigenvalues of the discriminant function were .51, .35, and .40, with the canonical correlations of .58, .51, and .54 at T2 and T0, respectively. The canonical coefficients of the discriminant functions are displayed in Table 3. The three discriminant functions were calculated as follows:


$$y_{\mathrm{T0}}\;=1.35\times \mathrm{weight}\;\mathrm{loss}+1.66\times \mathrm{reduced}\;\mathrm{energy}\;\mathrm{level}+0.35\times \mathrm{physical}\;\mathrm{function}-1.21,$$

**Table 3:** Summary of Stepwise Discriminant Analysis

Variable/Statistic	Standardized Coefficient of Loading		
	T0	T1	T2
Predictor			
Weight loss	.39		
Reduced energy level	.61		
IADL lifestyle		.65	
Physical function	.50		.67
Eating function		.65	.55
Canonical function statistics			
Canonical correlation (*Rc*)	.58	.51	.54
Eigenvalue	.51	.35	.40
Wilks’ λ	.66	.74	.71
χ^2^/*df*	43.68/3	27.61/2	31.88/2

*Note.* IADL lifestyle= activities of daily living domain in Kihon Checklist.

where weight loss and reduced energy level are binary variables: yes=1, no=0; physical is scored 0–5.


$$y_{\mathrm{T1}}\;=0.53\times \mathrm{IADL}\;\mathrm{lifestyle}+0.67\times \mathrm{eating}\;\mathrm{function}-1.17,$$

where IADL lifestyle is scored 0–5, and eating function is scored 0–3.


$$y_{T2\;}=0.48\times \mathrm{physical}\;\mathrm{function}+0.59\times \mathrm{eating}\;\mathrm{function}-1.56,$$

where physical function is scored 0–5, and eating function is scored 0–3.

The group membership results of the classification routine of the stepwise regression discriminant analysis are summarized in Table 4. At T0, 67.3% of prefrail/frail group participants (sensitivity) and 90.9% of robust group participants (specificity) were classified correctly. At T0, the discriminant function classified 79.1% of participants accurately, correctly identifying 67.3% of prefrail/frail group participants (sensitivity) and 90.9% of robust group participants (specificity). In terms of predicting the prefrail/frail and robust status of participants at T1, the discriminant function classified 71.6% of participants accurately, with a sensitivity of 64.6% and specificity of 78.7%. In terms of predicting the prefrail/frail and robust status of participants at T2, the discriminant function accurately classified 73.2% of participants, with a sensitivity of 69.4% and specificity of 77.1%.

**Table 4:** Results of the Classification Routine

Grouped Cases	Predicted Group Membership					
	T0 (*n*=110)		T1 (*n*=95)		T2 (*n*=97)	
	Robust	Prefrail/Frail	Robust	Prefrail/Frail	Robust	Prefrail/Frail
Robust	50 (90.9)	5 (9.1)	37 (78.7)	10 (21.3)	37 (77.1)	11 (22.9)
Prefrail/Frail	18 (32.7)	37 (67.3)	17 (35.4)	31 (64.6)	15 (30.6)	34 (69.4)

Note. T0=fifty robust and 37 prefrail/frail (79.1%) cases were correctly classified; T1=there were 37 robust and 31 prefrail/frail (71.6%) patients correctly classified; T2=there were 37 robust and 34 prefrail/frail (73.2%) cases correctly classified.

## Discussion

### Frailty Prediction

Frailty is a common problem among older individuals, with a variety of tools available for its assessment. In this study, combinations of existing frailty screening tool components were used, with results compared against those generated by the FP, the gold-standard benchmark, to explore the optimal composition of components for predicting frailty at different time periods. To the best of the authors’ knowledge, this was the first longitudinal study to employ the gold standard of frailty and incorporate seven domains from the KCL tool and three components from the SOF tool in the discriminant analysis and prediction of frailty states. The goal of this effort was to minimize the number of predictive factors needed to achieve sufficiently reliable and accurate assessment results.

Through longitudinal tracking, this study found that at baseline, weight loss, reduced energy levels, and physical function successfully predicted frailty status. At 6 months, the predictors shifted to the IADL lifestyle and the eating function. At 1 year, the predictors were physical function and eating function. The eating function served as a predictor of frailty at both 6 months and 1 year. Possible reasons for the shifting composition of predictors over time are that, although weight loss and reduced energy levels may indicate early frailty, as frailty progresses, more complex abilities are needed to maintain independent living (such as IADL lifestyle and the eating function). Discriminant analysis was used in this study to identify the most significant predictive factors of frailty among community-dwelling older adults across two follow-ups over a 1-year period. The discriminant analysis successfully classified 71.6%–79.1% of the participants in the robust and prefrail/frail groups.

### Predictors of Frailty: Weight Loss, Reduced Energy Level, and IADL Lifestyle

The state of frailty in the participants was shown to change across the study timeline. Kojima et al. (2019) conducted a meta-analysis, revealing that, after an average follow-up period of 3.9 years, 56.5% of subjects remained frail, 29.1% of subjects experienced worsening frailty, and 13.9% of subjects showed improvement. Weight loss, reduced energy level, and physical function were successfully shown to predict frailty state at baseline. The two components of the SOF tool, weight loss and reduced energy level, are derived from two of the five components of the FP: shrinking and exhaustion. This likely explains the prediction results of the frailty state at baseline in this study.

However, these factors were not significant predictors at the two follow-ups. Huang et al. (2020) found that better IADL among rural community-dwelling older adults is a protective factor against frailty. Yuan et al. (2022) found that IADL is a cross-sectional factor associated with frailty status, and that a decline in ADL/IADL can directly predict the worsening of frailty over a 1-year follow-up period. In this study, IADL lifestyle was found to be a predictor of frailty at the 6-month follow-up. This discrepancy with the findings of Yuan et al. (2022), which identified IADL as a predictor of frailty over 1 year, may be due to differences in study populations: The study by Yuan et al. included not only community-dwelling older adults but also nursing home residents. Despite these differences, both studies highlight IADL as a predictor of frailty. Fried et al. (2001) reported that 60% of frail individuals had difficulty with IADL, whereas only 27% had difficulty with ADL. Therefore, it may be inferred that IADL difficulties occur earlier than ADL difficulties in frail individuals. The findings of this study align with these results, showing IADL lifestyle to be a predictor at T1 (6 months), but not at T2 (1 year), when physical function is the predictor.

### Predictors of Frailty: Physical Function

Based on the discriminant functions at T0 and T2, physical function measured at baseline using the KCL tool predicts frailty outcomes at baseline as well as at 1 year, as confirmed using the FP. As no similar studies are available for comparison, the discussion in this paper focuses on the relationship between physical function and two components of the FP: slowness and weakness. Sewo Sampaio et al. (2016) reviewed the literature and found the physical domain of the KCL tool is referred to by various domain names, including “physical strength,” “walking status,” “physical,” “motor function,” “physical function(s),” “motor abilities,” and “musculoskeletal.” The physical function component of the KCL tool is measured using a self-report assessment of five mobility-related activities: the need for assistance to climb stairs and stand up from a chair, the ability to walk continuously for >15 minutes, fall history, and fear of falling while walking.

Kamasaki et al. (2023) demonstrated that prefrail older adults experience a decline in physical function at a rate 1.83 times higher than robust individuals. This decline is primarily attributable to difficulties faced in climbing stairs and standing up from a chair and a fear of falling. The European Working Group on Sarcopenia in Older People recommends that physical performance be assessed using the Short Physical Performance Battery, usual gait speed, and the stair climb power test. The Short Physical Performance Battery includes measurements of balance, gait, strength, and endurance. However, the stair climb power test is more complex as it involves measuring leg strength and performance, making it primarily suitable for research settings (Cruz-Jentoft et al., 2010). In this study, physical function was assessed using self-reported measures focusing on common mobility activities, including leg strength, balance, and overall physical performance. This approach aligns with Satake et al. (2020), who emphasized its efficiency and convenience compared to tools requiring multiple measurements, making it highly feasible for daily clinical practice and large-scale studies. One study found that frailty can reduce hip and knee flexion and knee and ankle internal rotation, leading to reduced hip adduction angles to compensate for walking stability. When enhancing balance and lower limb strength in frail older adults, special attention should be given to hip and knee flexion ranges, with corrections to walking posture made as necessary (Kong et al., 2022).

### Predictors of Frailty: Eating Function

The findings of this study, based on the discriminant functions at T1 and T2, indicate baseline eating function, as measured using the KCL tool, to be a predictor of frailty outcomes at 6 months and 1 year when assessed using the FP. Eating function as a predictor reflects self-reported difficulties in chewing hard foods, choking or coughing while drinking tea or soup, and experiencing dry mouth.

A study conducted by Japanese dentists showed reduced masticatory ability and other oral functions to be closely associated with prefrailty among community-dwelling older adults in Fukuoka Prefecture (Tani et al., 2022). Cruz-Moreira et al. (2023) found a significant correlation between oral hypofunction and frailty among female residents of care homes in Ecuador, with the strongest relationship observed between decreased swallowing function and frailty. The systematic literature review by Slashcheva et al. (2021) showed frailty to be significantly associated with masticatory ability and oral health. A meta-analysis by Kojima et al. (2022) reported that self-reported masticatory dysfunction increases the risk of frailty by a factor of 1.83. Furthermore, a systematic review of longitudinal studies on oral health and frailty by Hakeem et al. (2019) showed oral function, oral health problems, and symptoms of dry mouth to be significantly associated with frailty incidence. These findings align with the eating domain components identified in this study, emphasizing the relationship between frailty and aspects of oral health, including chewing, swallowing function, and dry mouth symptoms.

Parisius et al. (2022) conducted a scoping review that defined oral frailty as the age-related functional decline of orofacial structures. Tanaka et al. (2018) conducted a study on older adults in the Japanese community, finding that 16% exhibited oral frailty at baseline. Over the 2-year follow-up period, the risk of developing physical frailty increased by a factor of 2.4. This finding aligns with this study, which also found that oral function can predict frailty at 6 months and 1 year. Assessment components and domains taken from two existing frailty screening tools were used in this study. While both the SOF weight loss component and the KCL nutrition domain include monitoring changes in weight, only the SOF weight loss component was included in the baseline discriminant function. At the 6-month and 1-year follow-ups, the frailty predictors related to weight loss shifted to the KCL eating function. Possible explanations for the impact of oral health on frailty include its impact on associations with nutrition, food intake and choice, periodontal inflammation, and socioeconomic factors (Hakeem et al., 2019).

### Predictors of Frailty: Others

Vidal et al. (2020) conducted a cross-sectional study on 379 community-dwelling older adults and found inspiratory and expiratory muscle strength may be used to effectively discriminate among nonfrail, prefrail, and frail states. Although similar to this study, Vidal et al. (2020) used FP as the standard for measuring frailty; they used a manovacuometer to measure inspiratory and expiratory pressure, reducing the suitability of their approach to frailty screening in clinical settings. Discriminant analysis revealed that weight loss, reduced energy level, and physical function correctly classified 79.1% of group membership, with better prediction of the robust group than the prefrail/frail group at baseline. Discriminant analysis also showed that IADL lifestyle and eating function correctly classified 71.6% of group membership, with better prediction of the robust group than the prefrail/frail group at the half-year follow-up. Furthermore, discriminant analysis results showed that physical function and eating function correctly classified 73.2% of group membership, with better prediction of the robust group than the prefrail/frail group at the 1-year follow-up. The results of this study show that, while the accuracy of classification of the prefrail/frail group remained consistent over time, the accuracy of classifying the robust group decreased. However, across all three time points, the overall classification accuracy rate ranged from 71.6% to 79.1%, which is higher than that reported in the only similar study in the literature (Dayhoff et al., 1998), which correctly classified 65% of group membership. It is important to note that the Dayhoff study utilized a self-report survey that combined two measures, that is, ADL and the World Health Organization Assessment of Functional Capacity (WHOAFC), rather than the gold-standard FP tool used in this study, to measure frailty. The predictors of frailty identified in their study include dorsiflexion strength and balance, both of which require measurement tools to test. Thus, their proposed method is also not suited for use in routine clinical frailty screening.

The Integrated Care for Older People framework issued by the WHO in 2016 is used primarily in the measurement of intrinsic capacity (IC) deficits such as mobility, cognition, psychological capacity (depressive symptoms), vitality (malnutrition), and sensory capacity (hearing and vision; Banerjee & Sadana, 2021). These domains are generally measured individually and have yet to be incorporated into a comprehensive frailty screening score. Zhou et al. (2024) found the presence of at least three IC domain deficits to correctly identify frailty with a specificity of 83.5%. However, the ability to identify robust individuals achieved a sensitivity of only 38.6%. As cumulative IC losses increased, specificity for frailty increased and sensitivity declined. The main difference between Zhou et al. and this study is their use of the simple Clinical Frailty Scale rather than the FP as a frailty screening tool. In addition, the five IC domains in the Integrated Care for Older People framework require complex assessments, for example, the five-time chair stand test to evaluate locomotion, cognitive function assessment using a brief version of the Mini-Mental State Examination to evaluate word recall, and orientation to time and place.

### Limitations

This study has several limitations. First, most of the data used were self-reported, which may introduce bias and affect the results. Second, the sample was collected from a geographic area encompassing one-third of a county in central Taiwan, which may restrict the generalizability of the findings to other geographic areas. Third, tracking frailty state transition over a 1-year period may not be sufficient to reveal the long-term predictive power of the factors influencing frailty. Fourth, the psychometric properties of the SOF and FP tools are limited. According to a systematic review by Sutton et al. (2016), these tools demonstrate only criterion validity, which includes concurrent and predictive validity. Reliability has been reported only in Yang (2022). Due to the limited number of items, three from the SOF and five from the FP, the Cronbach’s α values were generally low.

### Recommendations

When conducting clinical assessments of older adults, it is essential not only to evaluate changes in disease status but also to consider functional abilities, which are crucial for daily living. Functional ability is closely related to intrinsic capacity. This approach supports the daily living activities of the elderly. The FP tool requires measurements of gait speed and hand grip strength as well as inquiries into activities performed over the past week, which can be time-consuming and inconvenient in clinical practice due to the need for space and equipment. Thus, the questionnaire-based SOF may be employed as an alternative to the FP in routine frailty screenings. To predict the frailty state of community-dwelling older adults over 6 months to 1 year, using the KCL as an alternative to the FP is suggested. Although SOF and FP are widely used to measure frailty and have demonstrated criterion validity, their reliability has not been reported. The low Cronbach’s α values observed in this study suggest further exploration is needed.

In addition, for rapid frailty screening in outpatient and community settings, the 13-item questionnaire of the KCL tool, focusing on physical function, IADL lifestyle, and eating domains, may be utilized. Identifying frailty predictors is crucial for the development of frailty prevention and intervention strategies. Most factors of influence, for example, weight, energy level, physical function, IADL lifestyle, and eating function, are modifiable and may serve as targets for interventions aimed at preventing or delaying frailty. The importance of eating as a predictor is particularly under-researched. Notably, Japan now includes “oral hypofunction” as a reimbursable diagnosis under its national health insurance program. Based on these findings, early identification using a simple questionnaire is beneficial in identifying and preventing frailty.

Nurses should be vigilant and provide education if community-dwelling older adults exhibit unintentional weight loss and reduced energy levels, as these may indicate a tendency toward frailty. Further assessment may include questionnaire-based evaluations of physical function, IADL lifestyle, and eating function. These evaluations are able to correctly classify frailty states up to 1 year in roughly 70% of community-dwelling older adults. By conducting preliminary assessments, nurses can identify abnormal cases and refer them to physicians, dietitians, rehabilitation therapists, and long-term care services to facilitate multidisciplinary interventions. Currently, discriminant functions require calculations via formulas. Future recommendations include developing a visualized app program based on these variables, validating its effectiveness in different settings, extending the follow-up period beyond 1 year, and further investigating the impact of eating function on frailty.

### Conclusions

Discriminatory analysis is a simple yet effective method for determining frailty status in community-dwelling older adults. By selecting and including the most important contributing SOF components and KCL domains, this method significantly reduces the number of variables needed to conduct accurate analyses. At baseline, weight loss and reduced energy level components of the SOF and the physical function domain of the KCL were identified as the most important contributing factors for predicting robust and prefrail/frail states in community-dwelling older adults. In addition, the IADL lifestyle and eating domains of the KCL tool at baseline were predictors of these states at the 6-month follow-up, while the physical function and eating domains of the KCL at baseline were predictors of these states at the 1-year follow-up.

This longitudinal study identified weight loss, reduced energy levels, physical function, IADL lifestyle, and eating function as the most significant factors at baseline for accurately classifying community-dwelling older adults as either robust or prefrail/frail over a 1-year follow-up period. Among these, the eating function was the most significant factor of influence on frailty state prediction accuracy. Health care providers and government agencies should integrate the findings of this research into long-term care policies to help prevent/delay the onset of frailty. Intervention measures should target significant contributing factors such as weight, energy level, physical strength, IADL lifestyle, and eating function, with special emphasis placed on the latter.
